# Molecular analysis of hepatitis B virus (HBV) in an HIV co-infected patient with reactivation of occult HBV infection following discontinuation of lamivudine-including antiretroviral therapy

**DOI:** 10.1186/1471-2334-11-310

**Published:** 2011-11-04

**Authors:** Andrea Costantini, Katia Marinelli, Giulia Biagioni, Alessia Monachetti, Monica L Ferreri, Luca Butini, Maria Montroni, Aldo Manzin, Patrizia Bagnarelli

**Affiliations:** 1 Clinical Immunology Unit, Department of Clinical and Molecular Sciences, Università Politecnica Marche, Via Tronto 10/a, 60020 Ancona, Italy; 2 Virology Unit, Department of Biomedical Sciences and Public Health, Università Politecnica Marche, Via Tronto 10/a, 60020 Ancona, Italy; 3 Section of Medical Microbiology, Department of Biomedical Science and Technology, Università di Cagliari, S.S.554, Bivio per Sestu, 09124 Monserrato (CA), Italy

## Abstract

**Background:**

Occult hepatitis B virus (HBV) infection (OBI) is characterized by HBV DNA persistence even though the pattern of serological markers indicates an otherwise resolved HBV infection. Although OBI is usually clinically silent, immunocompromised patients may experience reactivation of the liver disease.

**Case presentation:**

We report the case of an individual with human immunodeficiency virus (HIV) infection and anti-HBV core antibody positivity, who experienced severe HBV reactivation after discontinuation of lamivudine-including antiretroviral therapy (ART). HBV sequencing analysis showed a hepatitis B surface antigen escape mutant whose presence in an earlier sample excluded reinfection. Molecular sequencing showed some differences between two isolates collected at a 9-year interval, indicating HBV evolution. Resumption of ART containing an emtricitabine/tenofovir combination allowed control of plasma HBV DNA, which fell to undetectable levels.

**Conclusion:**

This case stresses the ability of HBV to evolve continuously, even during occult infection, and the effectiveness of ART in controlling OBI reactivation in HIV-infected individuals.

## Background

Occult hepatitis B virus (HBV) infection (OBI) is, by definition, characterized by infectious HBV DNA in liver, blood, or both, in the absence of hepatitis B surface antigens (HBsAg) [1]. Isolated anti-HBV core antibodies (anti-HBc) have been shown to be a predictive marker of OBI [2]. Isolated anti-HBc [3,4] and OBI are often seen in patients with human immunodeficiency virus (HIV) infection [5,6], where they are more prevalent than in non-coinfected individuals [7]. Reactivation of chronic HBV in presence of HBsAg has been reported in immunosuppressed subjects and in those with HIV infection following discontinuation of antiretroviral therapy (ART) [8,9].

There are few reports addressing OBI reactivation during HIV infection [10,11] and fewer still providing an extensive description of the molecular characteristics of occult HBV reactivation [12]. Nucleot(s)ide analogues (NA) lamivudine, emticitabine and tenofovir are known to be effective against both HIV and HBV, providing a unique opportunity to treat coinfected patients [13,14], but little information is available to establish whether resumption of ART for HIV/HBV coinfection may restore control of HBV replication after OBI reactivation.

## Case presentation

A 46-year-old woman with a 25-year history of HIV disease, who experienced two episodes of occult HBV reactivation after interrupting a lamivudine-containing ART regimen. At the time of the diagnosis of HIV infection (October 1985) she also tested negative for HBsAg and positive for anti-HBsAg and anti-HBc (table 1). Lamivudine-containing ART was started in November 1996 and repeatedly changed until August 2000, for a variety of reasons (table 2). In addition, the patient did not fully adhere to therapy, and complete suppression of HIV viremia was never obtained (not shown). In September 2000 she discontinued the ART treatment; in few months HIV RNA levels rose to more than 470,000 copies/ml and CD4+ T cell counts dropped to 9/mmc (table 2), leading to two episodes of esophageal candidiasis, interstitial pneumonia due to *Chlamydia pneumoniae*, and disseminated *Mycobacterium avium *infection (not shown). ART including lamivudine and tenofovir, was resumed in April 2002 and continued until September 2009. Improved adherence to treatment resulted in undetectable plasma HIV-RNA and high-level immune reconstitution (tables 1 and 2).

**Table 1:** Sequential serological, biochemical and virological findings in an HIV-infected individual with markers of prior HBV infection at baseline.

Characteristics	Date (month/year)															
	**10/85**	**11/87**	**10/00**^1^	**01/01**^1^	**05/01**^1^	**11/06**^1^	**01/07**^1^	**01/08**^1^	**05/09**	**12/09**	**02/10**	**02/10**	**03/10**	**04/10**	**05/10**	**07/10**

**HBsAg (IU/ml)**	NEG	NEG	0.00	0.00	0.00	0.00	0.00	0.00	0.00	--	168	183	132	66	0.1	0.0

**Anti-HBs (mIU/ml)**	POS	POS	<10	<10	<10	<10	<10	<10	<10	--	140	124	87	61	50	24

**Anti-HBc**	POS	POS	POS	POS	POS	POS	POS	POS	POS	--	POS	POS	POS	--	POS	POS

**Anti-HBc IgM**	NEG	NEG	--	--	--	--	--	--	--	--	POS	POS	POS	NEG	--	--

**HBeAg**	NEG	NEG	--	--	--	--	--	--	--	--	POS	NEG	NEG	--	--	--

**Anti-HBe**	NEG	NEG	--	--	--	--	--	--	--	--	POS	POS	POS	--	--	--

**HBV DNA (IU/ml)**	--	--	R (3)	R (7)	19	NR	NR	NR	NR	--	88,185	5,622	998	26	R (4)	NR

**AST (U/l)**	17	19	31	58	30	20	21	23	28	80	2,702	143	41	--	23	19

**ALT (U/l)**	14	16	35	68	37	14	14	14	19	111	2,577	306	42	--	17	21

**CD4+ T (cells/µl)**	891	883	48	29	48	478	580	516	513	304	291	--	--	--	423	333

**HIV-RNA**^2^**(copies/ml)**	--	--	147,018	222,107	146,707	<50	<50	NR	NR	80,558	--	--	734	--	NR	NR

**Antiretroviral therapy**	Naive	Naive	No^3^	No^3^	No^3^	Yes^4^	Yes^4^	Yes^4^	Yes^4^	No^5^	No^5^	Yes^6^	Yes^6^	Yes^6^	Yes^6^	Yes^6^

IU/ml = International Units/milliliter; mIU/ml = International milliunits/ml; U/l = Units/liter; NEG = negative; POS = positive; R = reactive below cut-off (<10IU/ml); HBV DNA calculated by the regression curve in brackets; NR = not reactive; -- = not determined.

^1^molecular and serological markers of HBV infection were retrospectively determined in February 2010 using automated Abbott HBV RealTime and Abbott ARCHITECT HBV assays, respectively

^2^HIV viremia was evaluated by Versant HIV RNA assay 3.0 (bDNA; Siemens Healthcare Diagnostics, Deerfield, IL) up to 01/2007 and by Abbott HIV RealTime since 01/2008.

^3^Previous antiretroviral therapy (Didanosine, Lamivudine, Nevirapine) was started on 04/2000 and interrupted on 08/2000; Lamivudine daily dose was 300 mg.

^4^Therapy was resumed in 02/2002 with different drug combinations including Lamivudine (300 mg daily) with or without Tenofovir (245 mg daily).

^5^Therapy (Lamivudine and Atazanavir) was interrupted on 10/2009.

^6^Therapy was resumed in 02/2010, including co-formulated Emtricitabine/Tenofovir (200/245 mg daily) plus boosted Darunavir.

**Table 2:** Changes in ART during follow-up and reasons for each change.

ART combination	Start	Stop	Reasons for the changes	Lowest number of CD4+ T cells/mmc	Highest HIV-RNA load (copies/ml)
Zidovudine, Lamivudine	11/1996	07/1997	Upgrade	147	--

Zidovudine, Lamivudine, Saquinavir	08/1997	02/2000	Poor adherence, virological failure	50	90,387

Stavudine, Lamivudine, Efavirenz	03/2000	04/2000	Skin rash	95	1,164

Stavudine, Lamivudine, Nevirapine	05/2000	08/2000	Patient decision	65	1,245

INTERRUPTION	09/2000	01/2002	--	9	472,578

Stavudine, Lamivudine	02/2002	04/2002	Upgrade	--	--

Stavudine, Lamivudine, Lopinavir/r*	05/2002	04/2003	Patient decision, diarrhea	74	<50

Tenofovir, Lamivudine, Indinavir/r*	05/2003	01/2009	Patient decision	245	<50

Didanosine, Lamivudine, Atazanavir	02/2009	04/2009	Simplification	516	<40

Lamivudine, Atazanavir	05/2009	09/2009	Patient decision	531	119

INTERRUPTION	10/2009	02/2010	--	291	80,558

Emtricitabine/Tenofovir, Darunavir/r*	02/2010	--	--	333	734

*r = boosted ritonavir.

Of note, since 1985 AST and ALT values were consistently in the normal range except on two occasions (May 1999, not shown, and January 2001, table 1), when slight increases were noted but not further investigated.

Another interruption of ART in October 2009 resulted in a sharp rise in aminotransferase levels to over 2,500 U/l in February 2010 (table 1), while CD4+ T cell counts fell from 531 to 291/mmc and HIV-RNA rose to >80,000 copies/ml. Antibodies against hepatitis C and hepatitis D virus were negative, but detection of serological markers of overt HBV infection (table 1) led to a diagnosis of OBI reactivation, which was further investigated at the molecular level.

**Figure 1:**
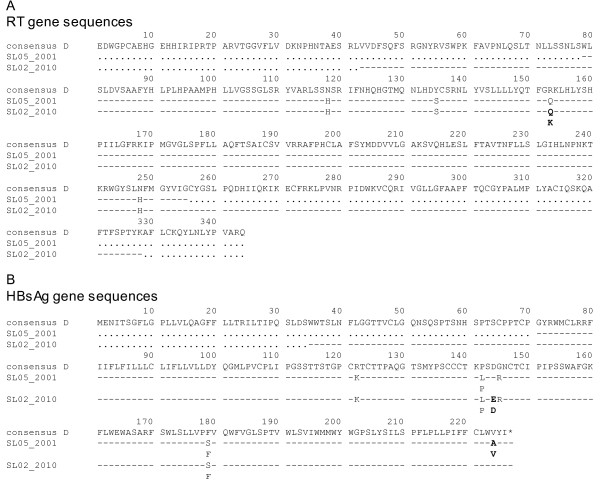
**Alignment of HBV genes**. Alignment of the amino acid (aa) sequences of the HBV polymerase (A) and surface (B) genes amplified from samples collected during two episodes of OBI reactivation. The genotype D consensus sequence is shown as a reference in the alignment performed by HIV-GRADE_HBV-tool. Dashes indicate identity to the reference, points indicate sequence gaps due to primer design. Substitutions and ambiguities indicating presence of quasispecies in the direct sequence are shown. Differences between the two sequences are in bold. * = stop codon; aa numbered according to Stuyver et al [26]. The two sequences have been submitted to the GenBank and assigned accession no. JF827279 (SL05_2001), JF827280 (SL02_2010).

A portion of the polymerase gene was sequenced using the PCR product obtained with primers P1 (forward outer = 5'-TCTAGACTCGTGGTGGACTTCTC) and P4 (reverse outer = 5'-TACAGAGAAAGGCCTTGTAAGTTG) which amplified an 880 bp fragment of HBV DNA from nucleotide 249 to 1128 (numbered according to an EcoRI site). This allowed analysis of mutations in the overlapping surface (s) and reverse transcriptase (rt) genes of HBV. As shown in table 3 and Figure 1, the patient harboured a genotype D (subtype D1) HBV strain and no NA resistance-associated mutations were selected in the polymerase gene. Interestingly, envelope escape variants were detected due to G145R, D144DE and P142LP mutations. The presence of HBsAg escape variants and the availability of seven frozen samples collected between October 2000 and May 2009 prompted a retrospective analysis for the molecular and serological markers of viral activity. Three of the seven samples collected during the aminotransferase "blips" between October 2000 and May 2001 exhibited very low-level viremia (table 1) using highly sensitive HBV RealTi*m*e (Abbott Laboratories. Abbott Park, IL; limit of quantification 10 international units per millilitre [IU/mL]), and serological analysis revealed isolated anti-HBc. Attempts to obtain an overlapping polymerase and surface antigen sequence was successful with the May 2001 specimen, using a 5× concentration and nested PCR with inner primers P2 (forward inner = 5'-TCCTGTCCTCCAACTTGTCCTG) and P3 (reverse inner = 5'-TGTGGCAATGTACCCCAACTTCCA) that amplified a 571 bp fragment internal to the P1-P4 product, from nucleotide 346 to 916. This sequence showed the G145R and P142L, but not the D144DE HBsAg escape variant. The sample also contained the V224AV quasispecies, which was subsequently undetectable (table 3 and Figure 1). Although we cannot exclude that the different quasispecies were present in 2010, our date indicate that during the course of treatment, the species with D144E probably became more dominant and the species with V224A decreased in proportion. Altogether these data can be regarded as signs of HBV evolution: however, we acknowledge that lacking of cloning analysis of HBV quasispecies is a possible limitation to these conclusions.

**Table 3:** Genetic variability of hepatitis B virus during occult infection.

Sequence information	
**Sample ID (HBV viral load)**	SL05/2001 (19 IU/ml)

**Genotype (subtype)**	D (D1 - 97.95%**^1^**)

**rt-HBV mutations: aa 79-255 ^3^**	N118H, Y135S, **R153Q**, N248H**^2^**

**s-HBV mutations: aa 71-227_stop _^3^**	R122K, P142LP, **G145R**, F179FS, V224AV**^2^**

**Escape mutations**	142L, 145R

**Resistance prediction**	None^4^

	

**Sample ID (HBV viral load)**	SL02/2010 (88,185 IU/ml)

**Genotype (subtype)**	D (D1 - 98.23%**^1^**)

**rt-HBV mutations: aa 43-330 ^3^**	N118H, Y135S, **R153KQ**, N248H**^2^**

**s-HBV mutations: aa 35-227_stop_^3^**	R122K, P142LP, **D144DE**, **G145R**, F179FS**^2^**

**Escape mutations**	142L, 144E, 145R

**Resistance prediction**	None^4^

^1 ^percent identity to the subtype as reported by HIV-GRADE_HBV-tool analysis (software available at http://www.hiv-grade.de/hbv_grade/deployed/grade.pl?program=hbvalg).

^2 ^mutated residues are defined with respect to the HBV genotype D consensus sequence, residues in bold indicate changes involving both rt and s genes.

^3 ^aa = amino acid; the range of sequenced amino acid residues covers the whole region of rt-HBV involved by antiviral resistance and the "a" determinant neutralizing antibody-binding domains of the s-HBV gene; aa numbered according to Stuyver et al [26].

^4 ^the resistance prediction and reference sequence were assessed by HIV-GRADE_HBV-tool coupled with the interpretative algorithm Geno2Pheno [hbv] (available at http://hbv.bioinf.mpi-inf.mpg.de/index.php).

The patient resumed ART to control HIV replication and counteract HBV reactivation. A regimen including emtricitabine/tenofovir plus boosted darunavir was prescribed in mid February 2010. After three months aminotransferase levels reverted to normal; follow-up of serological HBV markers showed the sequential disappearance of hepatitis B e antigen (HBeAg), IgM class anti-HBc, and HBsAg. Plasma HBV DNA gradually fell to undetectable levels (table 1).

## Conclusions

We describe the case of an HIV-infected patient with serological evidence of resolved HBV infection who experienced two distinct episodes of OBI reactivation after interrupting of lamivudine-containing ART regimens. In both cases, reintroduction of ART including HBV-active NA (lamivudine or emtricitabine/tenofovir) was followed by aminotransferase normalization and clearance of plasma HBV DNA. Different results have been reported in two HIV/HBV coinfected patients [10,12]. The reasons for the different outcomes are unclear, since only few cases of OBI reactivation among HIV-infected individuals have been reported to date. Emergence of HBV drug resistance following OBI reactivation was observed neither in our patient nor in the one described by Bagaglio and co-workers [12]; residual HBV replication before the overt phase of occult HBV reactivation was excluded in both patients. However, residual HBV replication and reactivation without prior HBV DNA positivity have both been described in HIV-infected patients [15,16].

Interestingly, OBI reactivation in our patient was treated with a drug combination including emtricitabine and tenofovir, while Bagaglio and co-workers used a single-drug tenofovir-based approach, and Chamorro and colleagues a scale up approach where tenofovir was introduced years after lamivudine [10]. Conceivably, different HBV-active regimens may determine different outcomes in OBI reactivation, and combination therapy may have in principle better chances to obtain long-term control of HBV replication than single-drug regimens, especially in the context of HIV-related immune suppression.

One of the most intriguing aspect of HBV pathogenesis is the accumulation, already in the early phase of infection, of covalently closed circular (ccc)DNA in the nuclei of infected hepatocytes, which is the basis for the establishment of viral persistence. cccDNA is the hepatic reservoir for HBV infection, found in all patients studied up to a decade after resolution [17] and in 50% of patients who had acute self-limited HBV infection 30 years previously [18]. Intrahepatic cccDNA can be considered as a candidate for OBI reactivation when immunological host conditions deteriorate.

In our patient molecular analysis of HBV DNA during reactivation disclosed an HBsAg escape variant, as shown by G145R and D144DE mutations in the HBsAg "a" determinant. Since HBV reinfection could not be completely ruled out, we analyzed the serum and plasma samples collected during the previous aminotransferase "blip", and found that the G145R variant was already present at that time.

Due to overlapping envelope and polymerase genes, the s-G145R variant is associated with the rt-R153Q mutation, which results in expression of an altered polymerase that is replication competent but seems to have reduced replication efficiency [19-21]. Among the few differences observed between the direct sequences of the 2001 and 2010 specimens, the s-HBV D144E variant seems to be important, since it determines a Q153K back mutation at the rt-HBV level. It is conceivable that rt-153K could be a secondary mutation connected with a fitness gain. Interestingly, coexistence of HBsAg and anti-HBs in 2010 specimens could be explained by presence of heterologous subtype-specific antibodies possibly directed against an HBsAg subtype different from HBsAg expressed in the past (primary) infection. At least five remarkable mutations were detected within the neutralizing epitope cluster of the surface protein in the genotype D consensus sequence, of which the D144E mutation has been described as a variant with "d" determinant opposite to the "y" determinant more frequently detected in genotype D [22]. Although it is impossible to establish whether this variant emerged in the 9-year interval between the two OBI episodes, or during the latest reactivation, our data indicate that HBV evolution may have occurred during OBI, and that this and some other mutations may have led to the induction of less effective antibody response against a reactivated virus.

OBI reactivation is a consequence of deteriorated immune function [23,24]; in our patient the overt phase of severe HBV reactivation arose in conjunction with decreased CD4+ T cell counts. However, clinical and biochemical levels reflecting severe HBV reactivation were not observed at other times when the patient's immune status was profoundly impaired (see table 2): this was not a surprise, in that a severe reactivation of hepatitis B requires a competent immune systems, and this is what happened during immune reconstitution in the absence of anti-HBV active drugs. Overall, it may be speculated that a qualitative, rather than a quantitative worsening of HBV-specific T cell responses may favour OBI reactivation in HIV-infected patients. Notably, persistence of functional T cell response has been reported in individuals with OBI and in HBsAg-inactive carriers [25].

The clinical significance of occult hepatitis is still a matter of controversy and further studies should examine the long-term clinical implications of occult HBV in HIV-infected patients.

In summary, this case stresses that ART for HIV/HBV coinfection can control OBI reactivation in HIV-infected individuals, and that in patients with previous HBV infection the withdrawal of anti-HIV drugs with activity against HBV must be approached with caution. Moreover, molecular HBV sequencing data support the hypothesis that HBV cccDNA, is not only the genetic archive for resistant viral genome emerging during treatment of chronic HBV, but also the genetic reservoir allowing continuous HBV evolution, through ongoing low-level replication, even during occult infection.

## Consent

The study was approved by the institutional ethical committee of the Università Politecnica delle Marche. Written informed consent according to Helsinki protocol was obtained from the patient for publication of this case report.

## Competing interests

The authors declare that they have no relationship (commercial or otherwise) that may constitute a dual or conflicting interest. This paper was not supported by a research grant and was generated as part of routine activities.

## Authors' contributions

AC, LB and MM: have been involved in patient clinical care and carried out the standard immunological assays. AC, KM, MLF, AMa and PB: have been involved in acquisition and interpretation of data. GB, AMo and PB: carried out the standard and specific virological assays and the molecular and genetic studies. AC and PB conceived of the case presentation and drafted the manuscript. AC, PB and AMa reviewed the manuscript. All authors read and approved the final manuscript.

## Pre-publication history

The pre-publication history for this paper can be accessed here:

http://www.biomedcentral.com/1471-2334/11/310/prepub
